# Supplementary material for: Outcomes of Beta-Lactam Allergic and Non-Beta-Lactam Allergic Patients with Intra-Abdominal Infection: A Case–Control Study
Source: Antibiotics (Basel). 2022 Dec 9;11(12):1786. doi: 10.3390/antibiotics11121786 (PMC9774689; doi:10.3390/antibiotics11121786)
Supplement: Supplementary file 1 [file antibiotics-11-01786-s001.zip › antibiotics-2026512-supplementary.pdf]

Supplementary Materials

Outcomes of beta-lactam allergic and non-beta-lactam allergic patients with intra-abdominal infection: a case-control study

Table S1. Inadequate antimicrobial therapy, therapeutic failures, healthcare-associated infections and antibiotic-related adverse events in beta-lactam allergic patients (BLA) treated with fluoroquinolones versus non-beta-lactam allergic patients (NBLA) treated with beta-lactam antibiotics (robustness analysis).

**Characteristic**	**BLA (*N* = 38)**<br>***N* (%)**	**NBLA (*N* = 38)**<br>***N* (%)**	**p-value**
Therapeutic failure	5 (13%)	5 (13%)	>0.9
Healthcare-associated infection	2 (5.3%)	5 (13%)	0.4
Adverse event due to antibiotics	0 (0%)	1 (2.6%)	>0.9
Empiric antimicrobial therapy	6 (16%)	7 (18%)	0.8
Directed antimicrobial therapy	5 (13%)	1 (2.6%)	0.2

BLA: beta-lactam allergic, NBLA: non-beta-lactam allergic

Table S2. Rate of therapeutic failure among beta-lactam allergic patients (BLA) and non-beta-lactam allergic patients (NBLA), and details of intra-abdominal infection type and disease severity.

**Characteristic**	**BLA (*N* = 43)**	**NBLA (*N* = 43)**	**p-value**
Therapeutic failure, *N* (%)	6 (14%)	6 (14%)	>0.9
- Cholangitis, *N*	2	2	
- Cholecystitis, *N*	0	1	
- Appendicitis, *N*	1	1	
- Diverticulitis, *N*	2	1	
- Peritonitis, *N*	3	3	
- Gram-negative bacteria, *N*	2	6	
- Gram-positive bacteria, *N*	2	4	
- ICU admission, *N*	5	4	
- Apache II score, median (IQR)	10 (8; 14)	14 (8; 21)	

BLA: beta-lactam allergic, NBLA: non-beta-lactam allergic, ICU: intensive care unit, IQR: interquartile range (25th and 75th percentile)
